# Bio-Strings: A Relational Database Data-Type for Dealing with Large Biosequences

**DOI:** 10.3390/biotech11030031

**Published:** 2022-07-30

**Authors:** Sergio Lifschitz, Edward H. Haeusler, Marcos Catanho, Antonio B. de Miranda, Elvismary Molina de Armas, Alexandre Heine, Sergio G. M. P. Moreira, Cristian Tristão

**Affiliations:** 1 Departamento de Informática, Pontifícia Universidade Católica do Rio de Janeiro (PUC-Rio), Rio de Janeiro 22451-900, Brazil; hermann@inf.puc-rio.br (E.H.H.); earmas@inf.puc-rio.br (E.M.d.A.); alexandreh@inf.puc-rio.br (A.H.); smoreira@inf.puc-rio.br (S.G.M.P.M.); ctristao@inf.puc-rio.br (C.T.); 2 Lab. Genética Molecular de Microrganismos, Fundação Oswaldo Cruz (FIOCRUZ), Rio de Janeiro 21040-900, Brazil; marcos.catanho@fiocruz.br (M.C.); antonio.miranda@fiocruz.br (A.B.d.M.)

**Keywords:** biological sequences, relational databases, string type, stored functions

## Abstract

DNA sequencers output a large set of very long biological data strings that we should persist in databases rather than basic text file systems. Many different data models and database management systems (DBMS) may deal with both storage and efficiency issues regarding genomic datasets. Specifically, there is a need for handling strings with variable sizes while keeping their biological meaning. Relational database management systems (RDBMS) provide several data types that could be further explored for the genomics context. Besides, they enforce integrity, consistency, and enable good abstractions for more conventional data. We propose the relational text data type to represent and manipulate biological sequences and their derivatives. We present a logical schema for representing the core biological information, which may be inferred from a given biological conceptual data schema and the corresponding function manipulations. We implement and evaluate these stored functions into an actual RDBMS for both efficacy and efficiency. We show that it is possible to enforce basic and complex requirements for the genomic domain. We claim that the well-established relational text data type in RDBMS may appropriately handle the representation and persistency of biological sequences. We base our approach on the idea of domain-specific abstract data types that can store data with semantically defined functions while hiding those details from non-technical end-users.

## 1. Background

There are many different approaches to storing and manipulating biological data. Relevant information inherent to this domain, such as nucleotides and their derivatives (proteins), are currently represented and interpreted as character string sets, with no explicit semantics. Moreover, even the derived information, such as the relationship between the sequences, which is part of molecular biology’s central dogma, is not appropriately considered. Additionally, there is no specific data structure for the storage and manipulation of biological data.

We represent all the genetic information of a living organism in its linear sequence of the four bases of DNA. Therefore, a four-letter alphabet (A, T, C, G) must encode the primary structure (i.e., the number and sequence of the 20 amino acids) of all proteins. The great difficulty in representing and manipulating biological sequences is related to its origin. Everything we know today about molecular biology is abstractions of how things are. Second, a biological sequence, taken in isolation, has no meaning of its own. The information is *hidden* in the set of letters that make up the sequence, requiring manipulation to extract such information.

Most approaches still persist data in files in some standard text format, e.g., the BLAST family [1] and SSEARCH [2]. Applications that use a Relational Database Management System (RDBMS) persist sequences in structures of type string or BLOBs or CLOBs (Character/Binary Large Objects) in their original form. This practice facilitates the loading of repositories from text files. However, a common solution still keeps very large character sequences, representing genomes in external files out of a DBMS control.

The use of a DBMS is a straightforward solution to manage large volumes of data. However, the available and usual data types are not ready for containing biological sequences adequately. The problem with treating a biological sequence as strings or BLOBs, used to store any data and files in general, is the loss of semantic information.

A biosequence or *biological string* has well-defined interpretations, e.g., nucleotide sequences, proteins, coding regions, etc. There are specific characteristics that differ from just a sequence of single characters, e.g., comparisons and similarity search are not simple pattern-matching programs. Neither are there appropriate mechanisms to answer queries related to identifying evolutionary relationships between genes—for example, homology, orthology, and paralogy, nor for functional (e.g., catalytic, regulatory) and structural (e.g., conserved regions, domains) relationships between genes.

Therefore, we might focus on the significant problem of representing and manipulating biological sequences directly on a DBMS. We will show that our data and functional requirements can be modeled directly in a robust and well-known relational system. However, there is no direct correspondence from very long biological sequences to relational data types. Moreover, those very long strings and sub-strings carry some semantics with them, and ideally, this should be taken into account.

Concerning any possible ADT-like (Abstract Data Type) solution, we usually have two alternatives: (i) the creation of a new data structure that addresses all requirements and defines a new way of storing and manipulating the data, or (ii) the extension of an existing data type, enforcing the list of requirements to match only the user needs.

If we think in terms of implementation, both alternatives have their pros and cons. On the one hand, creating a new type has the advantage of thinking and generating an appropriate structure and mechanisms for this new data type, which may have superior performance compared to an extended type. On the other hand, the effort to generate this new type is considerably higher: we need to create all the structures and mechanisms involved for storage and manipulation, which must run within an existing system. For the extended type, the scenario reverses. Depending on the data type used, the adaptation can be simplified, using any base already defined and developed.

In this paper, we propose the idea of creating functions associated with current data types available on a relational DBMS to obtain a more appropriate representation for very large bio-sequences, enabling their manipulation while keeping a biological interpretation. We will examine the string (or text) type and a set of biological-related functions that may answer both simple and complex queries, hiding implementation details from non-technical users.

Many existing relational and non-relational systems exist, e.g., GUS (https://www.cbil.upenn.edu/downloads/_local/sfischer/gus.html, accessed on 17 June 2022) and CHADO (http://gmod.org/wiki/Chado_Tables, accessed on 17 June 2022) that may be considered to deal with biosequences and many other genomic data types. Nevertheless, no details are given concerning the way they actually implement very large sequences representing biological information. It must be noted that we focus only on the biosequences type, not on complete biological or genomic database systems.

This work brings new unpublished contributions to the work presented in [3], including (i) a complete and extended conceptual model; (ii) explicit mappings for conceptual to relational transformations; (iii) a set of new basic and complex functions; (iv) an updated discussion on related works; and (v) results from a practical implementation within an actual relational DBMS.

## 2. Methods

In order to achieve our goals, we need to follow a complete relational database design path, starting from a conceptual data model and all the essential information that concerns biosequences and the corresponding applications. We avoid all non-biological terms and concepts to strengthen the existing semantics. Next, we give a closer look at the relational data model and a possible logical schema that one may obtain from the previous conceptual schema. At this point, we can propose a set of relational (stored) functions and procedures based on our string-type structure for complex and straightforward domain-oriented queries. We implement these functions using an actual relational DBMS (PostgreSQL) and run experiments on real datasets to check efficacy and efficiency.

### 2.1. Conceptual and Logical Modeling

We need to first discuss those basic and complex concepts that we represent in a pure Conceptual Data Model, such as the Entity-relationship one. Eventually, these will become relational database objects that we will take into account to detail and experiment our research work.

In nature, there are two types of nucleic acids: DNA (deoxyribonucleic acid) and RNA (ribonucleic acid). Analogously to a communication system, this information is kept inside the cell under rules that we call the genetic code. We see both DNA and RNA as a linear chain composed of simple chemical units called nucleotides in their primary structure. We may either refer to a nucleotide or base pair. The bases are Adenine, Guanine, Cytosine, Thymine, and Uracil, the first two are purines, and the last three are pyrimidines. In DNA, we find bases A, G, C, and T. In RNA, base U is observed instead of base T.

DNA molecules are made up of two strands which bond together, forming a helical structure, known as a double helix. The two tapes are merged by the stable connection between their nucleotide bases. Base A always binds base T, and base G binds base C. Thus, the nucleotide sequence in one strand ultimately determines the complete, double-stranded DNA molecule. It is precisely this property that allows for DNA self-duplication. Each strand of DNA has two ends, called 5′ and 3′, in an allusion to how carbon atoms are numbered in the sugar moiety that makes up each nucleotide and how they connect between nucleotides. The two tapes are antiparallel; that is, the tapes have a 5–3′ orientation opposite each other. The convention adopted worldwide to represent DNA molecules is to write only one of the strips in the 5–3′ direction.

### 2.2. CDs, ORFs and Proteins

A protein is generated from a gene, which is a region in a genomic sequence. A gene that encodes a protein produces a primary transcript that, after some processing, generates a mature transcript containing the protein-coding sequences (CDS). These sequences are formed by concatenating substrings containing information for proteins (exons) and untranslated regions (UTRs). ORFs may not be encoded in proteins. In this way, all coding sequences (CDS) are ORFs, but not every ORF encodes a protein.

**Figure 1:**
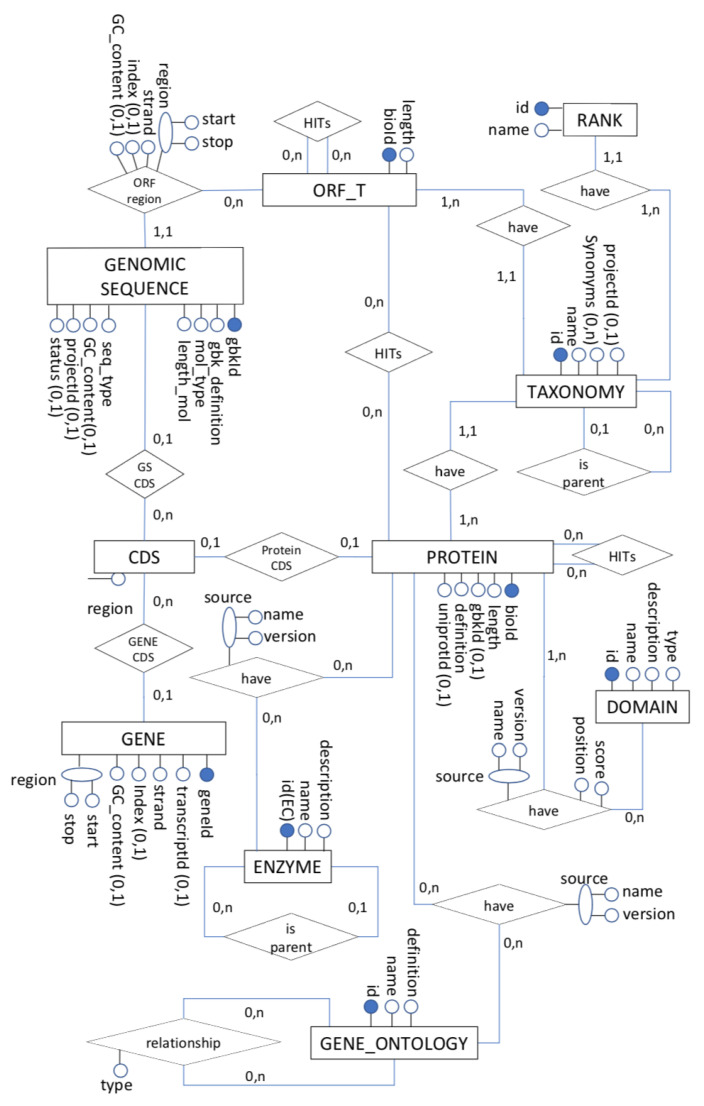
A Conceptual Biological Data Schema.

The protein entity (see Figure 1) represents the amino acid sequence of a protein with the nucleotide sequence of a CDS and the genomic sequence containing only an external reference to its transcription. Thus, the CDS is an entity whose primary property is to keep the relationship between the protein, gene, and genomic sequence entities. This is done by placing a given gene coding region (exons) in the coordinate system of the genomic sequence that contains it. Each exon in a gene corresponds to a subsequence CDS, defined by a starting and ending position mapped in a genomic sequence coordinate system.

### 2.3. Genes and Genomic Sequences

The nucleotide sequence of a gene that encodes a protein is part of a genomic sequence that will first be transcribed generating a pre-mRNA. This pre-mRNA molecule will be processed: the regions which will not be a part of the mature mRNA molecule (the introns) will be removed by a process called splicing. The mature mRNA molecule is now composed of exons; however, two of them (the 5′UTR and the 3′UTR) are not translated. Translated exons are composed of codons, which are nucleotide triplets that specify particular amino acids or act as signals for the beginning and end of the translation process. The reading and transcription of a gene generate the mRNA that eventually will be processed and transcribed into an amino acid sequence, which occurs in a specific direction in vivo (5′–3′).

The gene entity (Figure 1) has an identifier, and we will use the NCBI identifier (Entrez Gene (https://www.ncbi.nlm.nih.gov/Web/Search/entrezfs.html))—the *geneId*. We may define its corresponding region in the genomic sequence by a start and stop position, a sense of reading, a transcription identifier (from RefSeq), and the GC content. An $ORF_{T}$ amino acid sequence is analogous. It relates to the genomic nucleotide sequence through an $ORF_{region}$ delimited by a start and stops position within the genomic sequence, with the RefSeq identifier of the genomic sequence, the reading direction, its position concerning its neighbouring gene, and the sequence itself.

A genomic nucleotide sequence, derived from a RefSeq, refers to the genes containing CDSs that code for the protein amino acid sequence. These genomic sequences have a status that refers to the current stage of the sequencing research project. The possible values are:

- *Complete*, which typically means that each chromosome is represented by a single scaffold of a very high-quality sequence;
- *Assembly*, which typically means that scaffolds have been built not at the chromosome level or are of a low-quality sequencing project; and
- *In Progress*, indicating that both the sequencing project is in the pre-assembly or the completed (assembled fragments) strings have not yet been submitted to public databases such as the GenBank or EMBL.

The Genomic Sequence entity (Figure 1) has a RefSeq identifier, definition, and the length of the sequence, the type of organic molecule (DNA or RNA), status, type of sequence (chromosome, organelle, plasmid), an optional identifier of the respective genome project, GC content, and an identifier of the original taxon.

### 2.4. ORFs, Proteins and Taxonomic Classification

The taxonomy of organisms is an essential organizing principle in the study of biological systems. Inheritance, homology by common descent, and the sequence and structure conservation are all central biological ideas directly related to any group of organisms’ evolutionary history. A taxonomic classification follows a tree hierarchical structure. We call this path from the tree root node to any other particular taxon a *lineage*.

Regarding similarity information, there are three possible combinations of hits involving translated ORFs and proteins (Figure 1): (i) ORFs x ORFs; (ii) proteins x ORFs; and (iii) proteins x proteins. The minimum cardinality for all relationships is zero if the comparison does not generate significant hits. The maximum cardinality is n, as there may be several significant hits between the comparisons.

The translated amino acid sequences (ORF) are represented by another entity—$ORF_{T}$—because they do not have a previous identifier. Information about these strings includes the reference to the original organism, location, and size. There are also three distinct types of relationships between hits, proteins, and translated ORFs:

- hit_OO—result of the comparison between translated ORFs;
- hit_OP—result of the comparison between ORFs translated with proteins derived from SwissProt. Proteins derived from RefSeq were not used in the comparison process with the translated ORFs;
- hit_PP—result of the comparison of RefSeq proteins with RefSeq and SwissProt proteins.

These relationships (Figure 1) have attributes that specify the comparison process’s result, based on the information obtained using the Smith–Waterman (SW) Alignment algorithm [4]. These are query gi, subject gi, SW score (gross score of the comparison), bit score (score normalized), e-value (alignment significance), % identity, alignment length (alignment size), query start, query end, subject beginning, subject end, query gaps, and subject gaps.

### 2.5. Biological Annotations

It should be noted that our biological conceptual scheme also includes information related to protein annotations. These correspond to an annotation is a process of assigning predicted biological functions, and structural characteristics, to raw data, e.g., to the protein’s primary sequence [5].

It is noteworthy that the prediction of cellular functions (structural, enzymes, transporters, signalers, etc.) is essentially hypothetical. Most of these possible functions are attributed by in silico analysis, and only a small fraction of these predicted proteins had their functions confirmed by laboratory experiments. This shows the importance of wet labs validation combined with “dry” software simulations.

Our conceptual scheme (Figure 1) includes the following information regarding the annotations:

*Enzymes and Metabolic pathways:* KEGG represents a group of organic substances of nature, typically protein—there are also enzymes made up of RNA, ribozymes—with intracellular or extracellular activity, which have catalytic functions [6].

*Domain:* Pfam is a family database and protein domain, represented by a collection of multiple alignments of Markov sequences and models [7].

*Ontology:* Gene Ontology (GO) is a domain ontology, formed by three categories of concepts, namely: *Molecular Function*, *Biological Process*, and *Cell Component*. It aims to produce a controlled vocabulary that can be applied to all organisms, to represent knowledge in describing genes and protein roles in cells [8].

### 2.6. A Logical Relational Model

Once one has a conceptual schema, we must think about a corresponding logical schema looking forward to actual manipulations for biological applications and users. A straightforward mapping from our conceptual schema may be quickly obtained considering standard rules for transformations to the logical–relational model.

**Figure 2:**
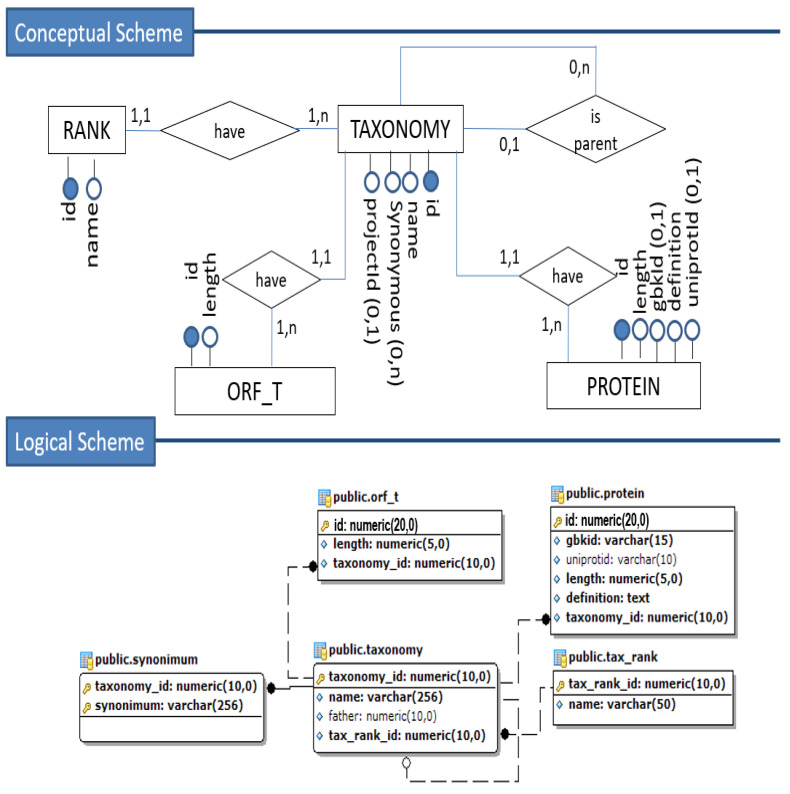
Conceptual to Logical Mapping—part 1.

**Figure 3:**
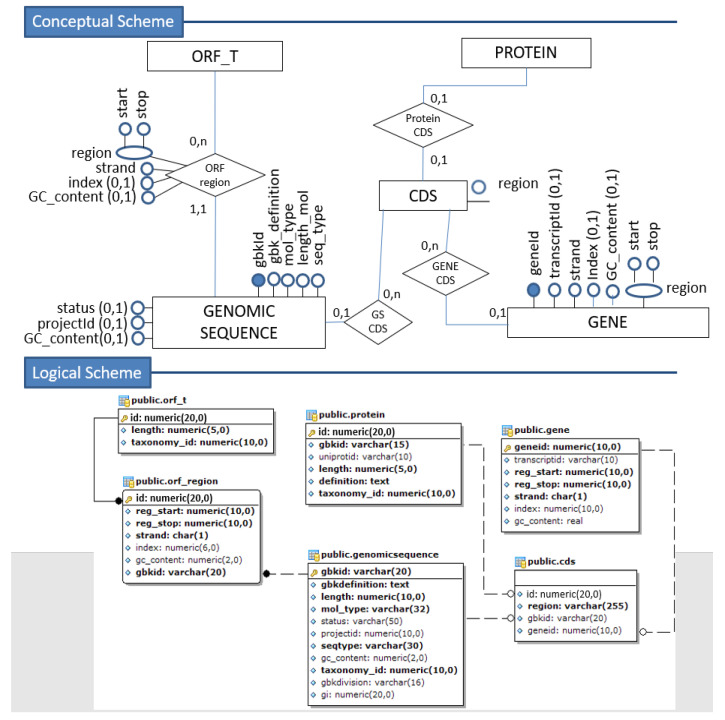
Conceptual to Logical Mapping—part 2.

**Figure 4:**
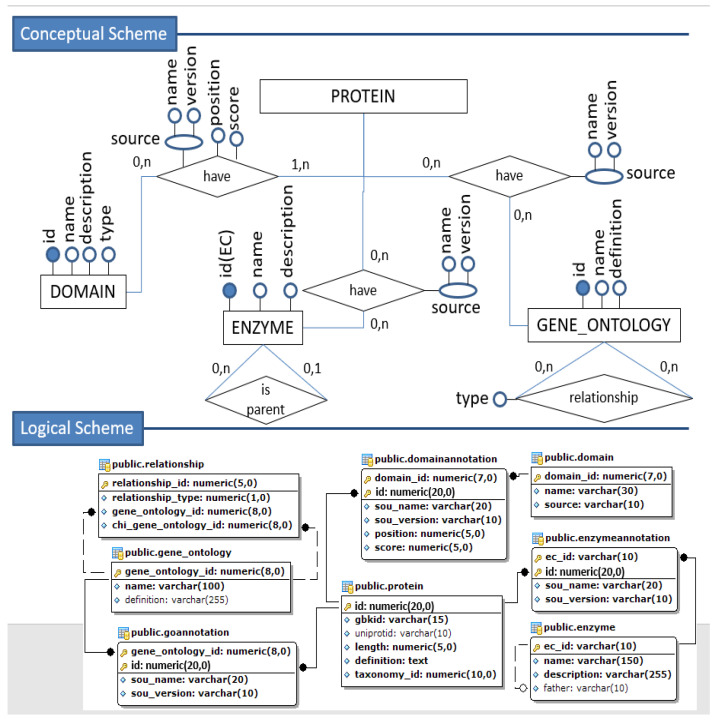
Conceptual to Logical Mapping—part 3.

The set of Figure 2, Figure 3 and Figure 4 illustrates the transformation process from a fraction of the conceptual schema to the corresponding relational (tabular) logical schema.

Based on our conceptual scheme, we observe that the relationship between proteins and their annotations is of the “many-to-many” type. Therefore, the mapping for a logical–relational scheme obtains by creating an intermediate table for each relationship. As the primary key, we will have the composition of the primary keys of the other two tables involved, originating from basic entities, and can also add the attributes that identify that relationship.

As a result of the association of the protein entity (and corresponding relation) between the *domain*, *enzyme*, and *gene ontology* entities, we get the *domain-annotation*, *enzyme- annotation*, and *go-annotation*, respectively. Finally, the self-relationships of the entities *enzyme* and *gene ontology* were mapped, generating the respective foreign keys in their tables.

*Enzyme*: as a “one-to-many” self-relationship, the same mapping used for the taxonomy is applied. The Enzyme table receives a new attribute called “father” (foreign key for the enzyme), which can be null, by definition and also according to the cardinality present in the conceptual scheme (“0,1”).

*Gene ontology*: the self-relationship of gene ontology is of the “many-to-many” type. For this reason, a specific table named *relationship* was created. The relationship type attribute (relationship-type) and a sequential attribute called relationship-id (that plays the primary key role) were added to the ontology table. The need to create this attribute to identify the primary key and the non-use of the ontology reference attributes as a key is because there are different correspondences for the same relationship. This situation is illustrated in Figure 4.

Through proper mapping, we obtained a relational logical scheme from the conceptual model (Figure 1), using the set of traditional transformation rules and specific adjustments due to some performance issues. We may follow analogous steps to reach the complete relational schema.

## 3. Results

We claim that it may not be a problem to manage biological sequences considering the relational data model and an RDBMS. Instead, the lack of semantics in the existing data structures is an issue. We propose and discuss in this paper the idea of a *bio-string* type using an extension of the widely present *text* or *string* type. The main reason is the complexity of storing and representing biological sequences in BLOB structures concerning expressiveness. As BLOBs are designed to hold any possible data, there are no appropriate access and manipulation mechanisms.

The string type structure has a well-defined storage pattern and mechanisms for accessing and manipulating data. Common string functions such as *lower*, *upper*, and *convert* do not make any sense for a biological applications. Nevertheless, if we use the string storage structure for biological sequences, we must create or rewrite functions or operators specific to the molecular biology domain.

### 3.1. Bio-Strings in Action

We present a straightforward case study with the PostgreSQL (https://www.postgresql.org/, accessed on 17 June 2022) relational DBMS for prototyping the idea proposed in this paper.

More than representing and relating the concepts involved in the domain, the relational (logical) scheme must answer conceptual and theoretical questions about the represented objects. We present some analysis queries concerning our biological (conceptual and logical) scheme categorized into two groups: basic and complex.

To check if the schema can provide the information to the suggested analysis, we can make queries using standard Structured Query Language (SQL) considering our relational database implemented with the PostgreSQL relational DBMS. It is worth noting that without using the proposed modeling solution, even the queries considered more straightforward would not be easily answered.

### 3.2. Basic Queries

This group refers to analysis where the information concerns only one or more objects’ statistical data—for example, accounting for occurrences or tuples of a given object, or between objects that are related to each other. From a biological point of view, these are fundamental issues that provide valuable information.

Although the base composition varies from one species to another, the Adenine amount is always equal to that of Thymine (A = T). The number of Guanine and Cytosine bases is also the same (G = C). Consequently, the total amount of purines is equivalent to pyrimidine (i.e., A + G = C + T). On the other hand, the AT/GC ratio varies considerably between species.

**Figure 5:**
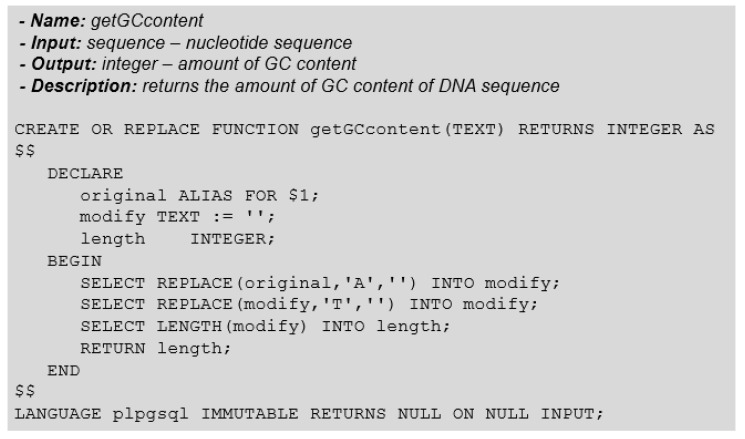
GC Content Relational Function.

Knowledge about the GC content of a DNA sequence is vital for determining the physical properties of DNA. The function for obtaining the GC content returns the number of bases G and C of a given input sequence. Unlike the other functions, which had to go through the nucleotide sequence to obtain the desired information, the *getGCcontent* function (Figure 5) had its implementation simplified using predefined functions in the relational TEXT type, such as the *replace* function.

For the construction of the getGCcontent function, we use the replace function to return the occurrences of bases A and T with an empty character (“ ”), eliminating the sequence’s bases. Subsequently, to obtain the GC content from the biological sequence, we must only get the resulting sequence’s size, which now has only bases G and C.

In the DNA nucleotide chain, a set of three nucleotides corresponds to a triplet. Through the transcription process, as mentioned before, some of these DNA triplets are converted into codons in the mature mRNA, and now they specify amino acids. The function that transforms a DNA sequence into an mRNA sequence is the transcript function. Its implementation is based on the getGCcontent function, requiring minor changes. It then migrates to the cell’s cytoplasm, where it binds to a ribosome and a carrier RNA molecule. The ribosome connects free amino acids to form the proteins through the *translation process*, using the genetic information of the individual’s DNA with the RNA molecule.

**Figure 6:**
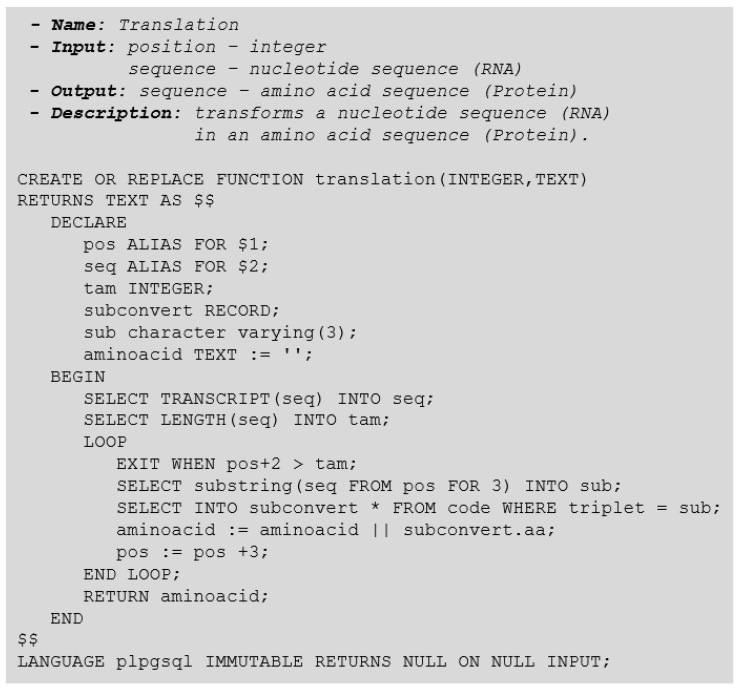
Translation Function.

To carry out the translation function, we must go through the nucleotide sequence of an mRNA molecule and convert them into amino acids at each sequence of three bases. The translation function depends on a translation table of the genetic code. Two approaches are possible: (1) storing the translation table information in an auxiliary storage structure, or (2) inserting the mapping of nucleotide sequences into amino acids directly into the function body. As a matter of simplification, we have used the first alternative (see Figure 6).

An ORF (Open Reading Frame) is a nucleotide sequence in a DNA molecule that can encode a peptide or a protein. Every protein originates from an ORF, but not every ORF originates a protein. An ORF is bounded by the AUG initiation codon, which encodes the amino acid Methionine (Met), indicating where the protein’s amino acid sequence encoding begins. The termination codons (UAA, UGA, and UAG) act as signals, indicating that the amino acid sequence destined for that protein ends there. In this way, all proteins begin with the Met amino acid. An ORF that does not have the identified protein product is called URF (unidentified reading frame).

**Figure 7:**
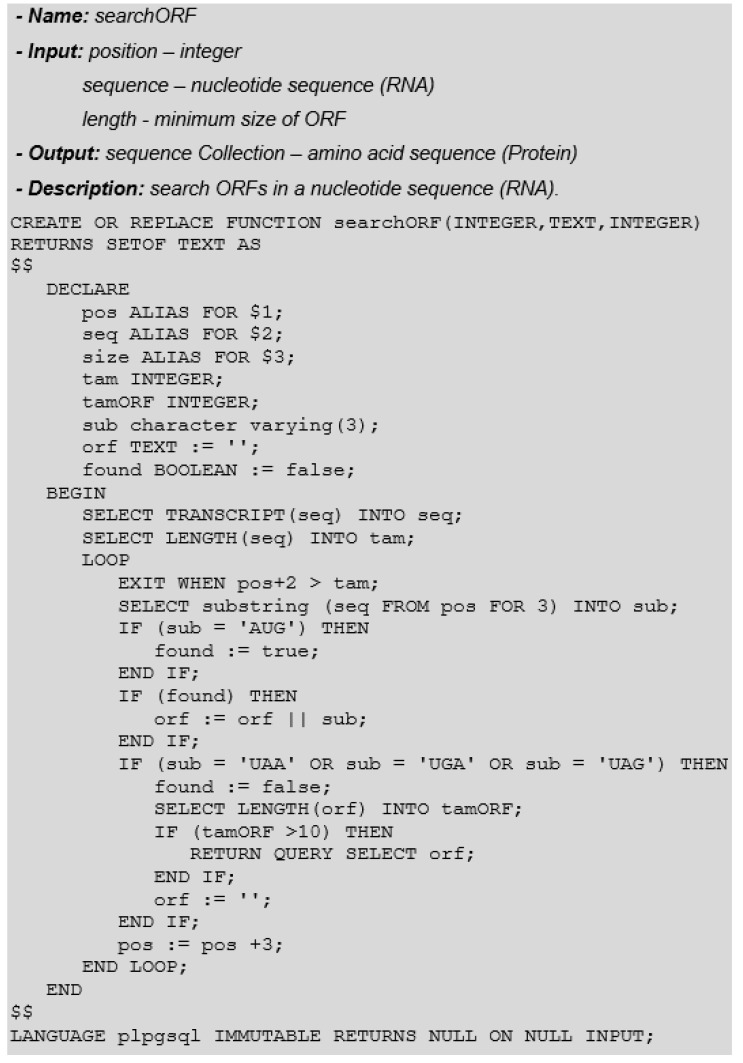
Search ORF Function.

As with the translation function, we must inform the reading frame (1, 2, or 3) to correctly identify codons. Another parameter is the minimum size of the ORF. Like translation, the search for ORFs is performed on an RNA sequence. To avoid user errors (e.g., use a DNA sequence instead of RNA), the function transcribes the input sequence before performing the search. Figure 7 illustrates this function.

To obtain the number of genomes that belong to a taxonomic group, we must first know the particular taxonomic group’s identifier. Next, we must obtain all taxonomy identifiers (taxonomy-id) included in the chosen taxonomic group.

Obtaining this type of information requires a process similar to searching tree data structures. In relational DBMSs, we can perform this query by applying recursion, with the “WITH” clause and the so-called Common Table Expressions (CTE).

**Figure 8:**
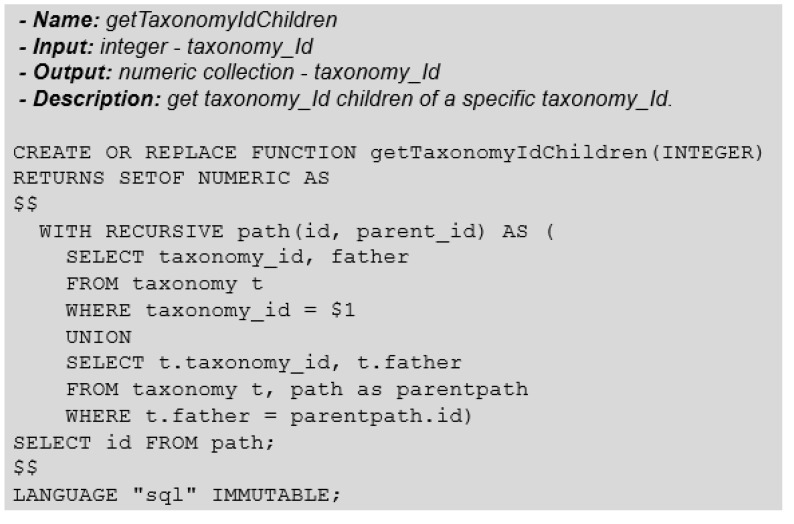
Function: GetTaxonomyIdChildren.

Now that we can obtain the child nodes of a tree data structure, we may create a function to facilitate the search process for the descendants of any node in the tree (Figure 8).

**Figure 9:**
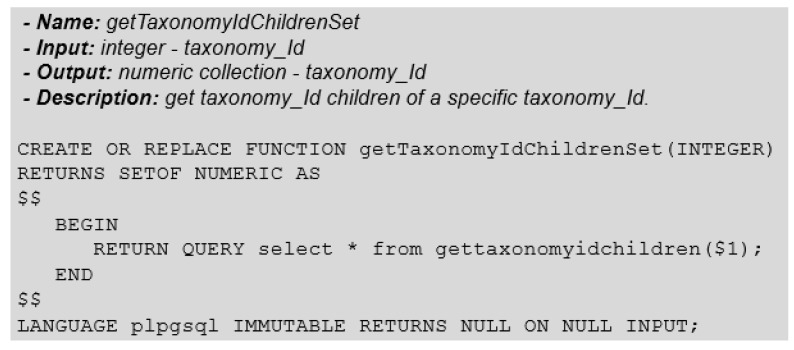
Function: GetTaxonomyIdChildrenSet.

In the case of PostgreSQL and its PLPGSQL language environment, we have to deal with some incompatibility problems, as is the case with the output type of the stored function in Figure 8 with the data types handled by a PLPGSQL function. In this way, we had to implement a new auxiliary function. It works as a type cast converter to be reused by other PLPGSQL functions (Figure 9).

### 3.3. Taxonomies

Next, we count the number of species belonging to this group with original genomic sequences (of the compared proteins) in the Genomic Sequence entity. The result may be obtained by comparing the genomic sequences’ taxonomy-id with the corresponding id for the species belonging to the desired taxonomic group.

**Figure 10:**
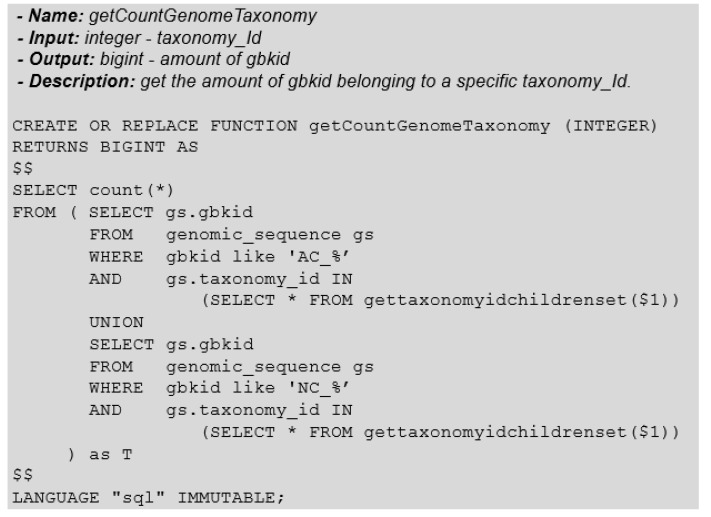
Function: GetCountGenomeTaxonomy.

Reiterating the focus on ADTs to facilitate non-specialized end-users interested in these searches, the query that returns the number of genomes belonging to a taxonomic group can also be made available as a function (Figure 10).

Taking a closer look at the function that returns the number of genomes of a given taxonomy, we realize that the query filters the genomic sequences (*gbkid*—genbank identification). We may justify this filtering action because the genomes are represented by:

*AC_*: Prefix used for genomic molecules that reflect an alternative annotation or assembly. Mainly used for prokaryotic and virus records.

*NC_*: Represents complete genomic molecules, including genomes, chromosomes, organelles, and plasmids.

*NG_*: Incomplete genomic region, which supports the NCBI genome annotation pipeline. It represents non-transcribed pseudogenes, or larger regions representing a grouping of genes that are difficult to annotate using automatic methods.

*NT_* and *NW_*: assemblies of intermediate BAC genomes and (or) complete genome.

*NZ_*: a collection of complete genome sequence data in a research project. Memberships are not tracked between releases. The first four characters that follow the underscore (e.g., ‘ABCD’) identify the project.

*NS_*: Genome records representing an assembly that does not reflect the structure of a concrete biological molecule.

Therefore, we are only interested in the practice with the genomic sequences with particular identifiers, precisely, “AC_” and “NC_”, as we see in Figure 10.

### 3.4. Proteins and Taxonomic Groups

Like the previous query discussed, we only have to count all proteins originating from the genomes belonging to a given taxonomic group. However, we facilitate this process since our biological design modelling (conceptual and logical) enables some exciting abstractions.

In our proposal, the conceptual scheme and, consequently, the relational scheme derived from it, tries to be as faithful as possible to the concepts used in molecular biology theory. However, to satisfy some practical needs, the scheme needed some adaptations. For example, in theory, every protein originates from a genome. However, proteins are often sequenced without knowing the source genome. In this way, the Protein object (table or entity) has the taxonomy-id information as an attribute because, even though it does not know the genome, the sequenced species are known.

**Figure 11:**
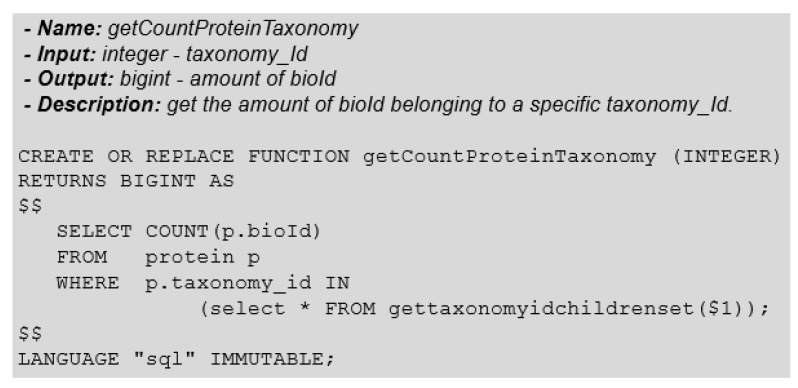
Function: GetCountProteinTaxonomy.

Figure 11 shows the function that returns the number of proteins that belong to a taxonomic group.

**Figure 12:**
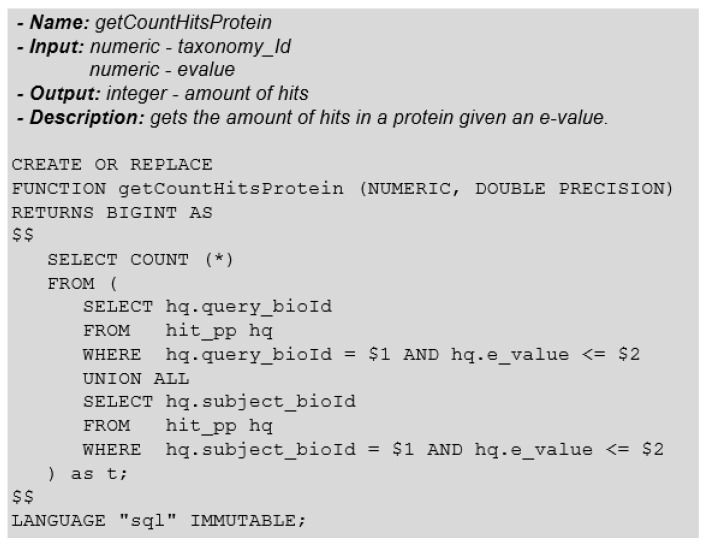
Function: GetCountHitsProtein.

This type of information is beneficial for the analysis and discovery of new proteins. The number of hits a protein obtains in the alignment process can be obtained by the number of occurrences of this protein in the table called *hit-pp*. Note that an occurrence can be either in query-id or subject-id. Besides, we should only consider hits that are below a certain e-value function parameter. This procedure can be implemented as a relational DBMS stored function (Figure 12).

With minor modifications we can obtain a broader range of information. If it is of interest to the user, we could check the number of hits of a protein, taking into account only ORFs (proteins and ORFs). In the first case, instead of searching the *hit-pp* table, we would use the *hit-op* table and compare only the subject-id that represents the protein. In the second case, we should add the search performed in both *hit-pp* and *hit-op* relations.

Another way to extend this query would be to use, as a filter criterion, not only the e-value but also all the attributes resulting from the comparison process, existing in the hit tables: SW score (Smith–Waterman score), the bit score, the percentage of identity), just to mention a few.

### 3.5. Complex Queries and Functions

This group of queries involve more profound concepts and knowledge of molecular biology. Taking into account the conceptual scheme of Figure 1, which was structured exclusively based on the concepts of molecular biology, without mentioning technological issues, we also facilitated the process of obtaining this type of information within our logical schema.

The following are some examples of functions that represent slightly more complex queries for obtaining biological information, such as discovering unique genes and homologous genes (orthologous and similar).

### 3.6. Unique Genes

Unique genes are genes found exclusively in certain groups of organisms. Different groups of organisms have different genes that we have not found in other groups. The discovery of unique genes is significant for investigating diseases and specific characteristics of individuals. In the process of searching for unique genes, we must find proteins from a complete proteome of a given species, which are not similar to any of the proteins of another proteome of a different species. For example, we must identify proteins belonging to complete proteomes of different species concerning this genus at the genus level, which may be similar within the taxonomic group, but with no biological similarity with proteins outside the corresponding genus.

**Figure 13:**
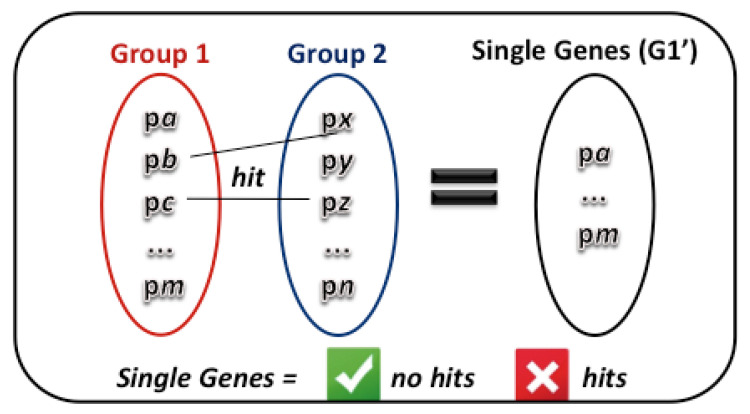
Set Theory: Identifying unique genes.

Figure 13 illustrates, using operations from set theory, what the expected result should be. A step by step procedure to achieve the desired goal is as follows:

1. Define the selected organism’s taxonomy-Id;
2. Search for the genomic sequences in the GenomicSequence object that belong to the selected taxon. As a result, we will have a set of genomic sequences;
3. For each element of the set of genomic sequences we must find the set of related proteins;
4. Finally, among this set of proteins, we must select those that do not hit with proteins from another group.

Once again, we can take advantage of the abstractions present in our conceptual scheme and reflected in the relational scheme. As we have taxonomy-id in proteins, we can directly search for proteins without consulting the genomic sequences beforehand, thus avoiding step 2 above.

**Figure 14:**
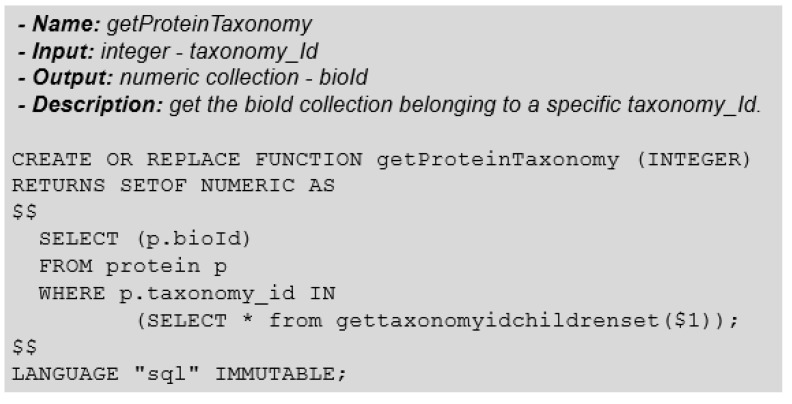
Function: GetProteinTaxonomy.

First, we need to find the set of proteins related to the organism in question (group 1). For this, we will use a function similar to *getCountProteinTaxonomy*. Instead of returning the number of proteins related to a taxonomy-Id, the only difference is that the function must return a list of the proteins with their respective identifiers. Figure 14 represents this function.

The next step is to find the set of proteins related to the other organism (group 2), using the getProteinTaxonomy function and passing the taxonomy-Id of this other organism as a parameter.

**Figure 15:**
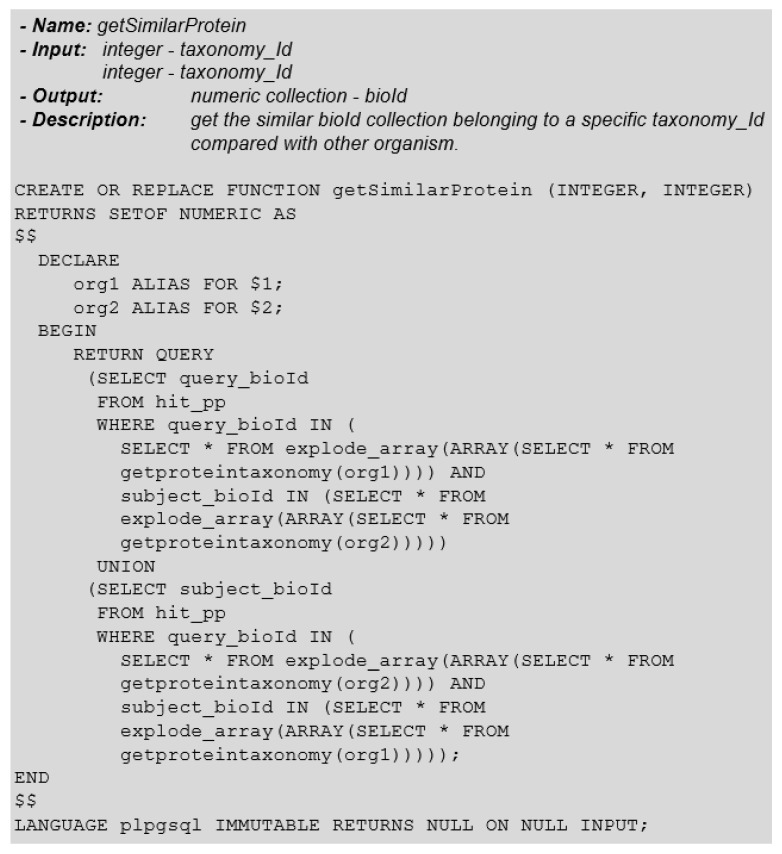
Function: GetSimilarProtein.

Then, we need to identify whether any proteins of the research organism (group 1) are similar with the proteins of the other organisms (group 2), thus generating a third group (group 3). For this, we must analyze the *query-id* and *subject-id* attributes of the protein similarity table (hit-pp) to find occurrences of records involving the relationship between these two groups. Figure 15 shows this function definition, responsible for this protein similarity procedure.

**Figure 16:**
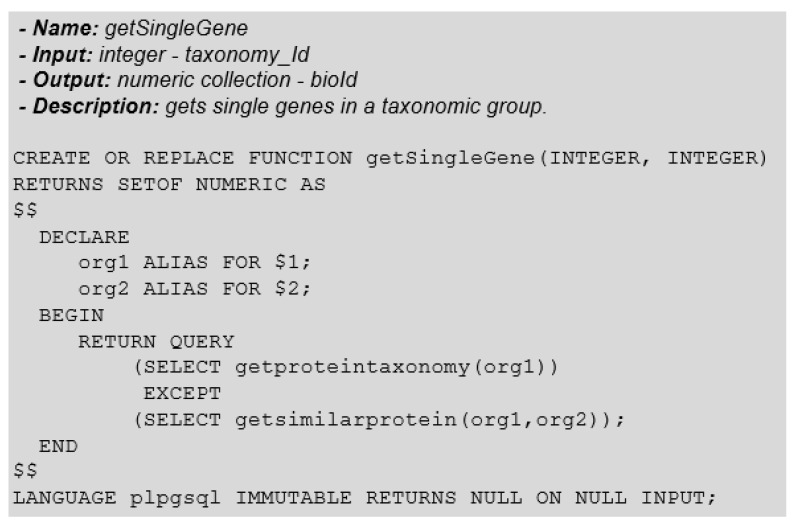
Function: GetSingleGene.

Finally, we must verify which proteins did not align with any other protein in the neighbouring group. For this, we need to eliminate from the set of proteins of the source organism (group 1) the proteins that have similarity with the other organism (group 3). In Figure 16 we give the PLPGSQL code for the function that represents the identification of unique genes based on our biosequence text-type.

It must be noted that this *GetSingleGene* function, as well as those auxiliary ones, can be extended using the desired e-value information, or some other hit-pp attribute, as an input parameter, to restrict the search and obtain more accurate and precise results.

### 3.7. Orthologs and Paralogs

Homology is the biological study of the similarities between different organisms’ structures with the same ontogenetic and phylogenetic origin. Such structures may or may not have the same biological function. All genes, in one way or another, can be considered homologous. We can trace the evolutionary history of all organisms down to the common ancestor of any living beings.

The homology of genes can be of two forms:

- *Orthologs*: genes found in different taxa that, when compared, are traceable to the events that led to speciation;
- *Paralogs*: genes in the same or different taxa, related to occurrences of gene duplication.

**Figure 17:**
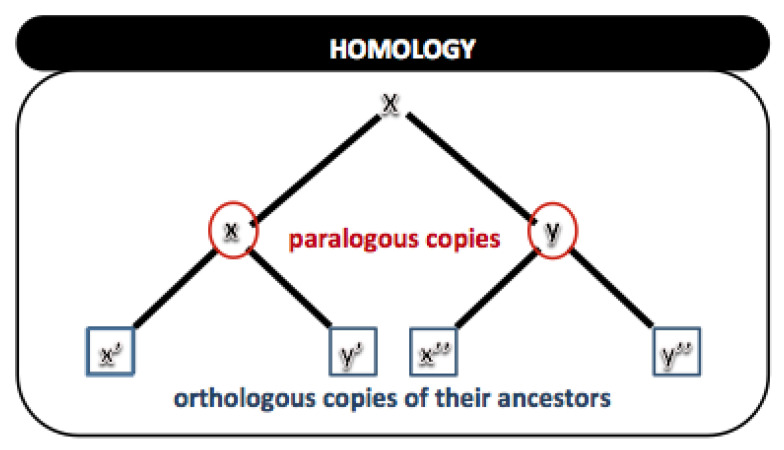
Homology Example.

Orthology and paralogy are key concepts in evolutionary genomics [9]. Figure 17 shows an example of homology.

Let it be a gene “x” that, from a gene duplication event, starts to present two copies, “x” and “y”. These are called paralogs, copies of the same x gene. Assuming that, over time, this population goes through a speciation event, the two copies will evolve independently in the two species, accumulating unique substitutions and, thus, differentiating. Therefore, the resulting copies x′, x″ and y′, y″ will be orthologous copies. All copies, either orthologs or paralogs, are considered homologs, and they usually share a high degree of similarity between their sequences.

In homology studies, the objective is to identify proteins from a complete proteome of a given organism that are evolutionarily related to other proteins of different proteomes. The identification of homologous genes follows the same logic as single genes. The goal is to find those proteins with no similarity in single genes, unlike in homology studies where searches for evolutionarily related proteins use biological similarity search as a defining criterion.

**Figure 18:**
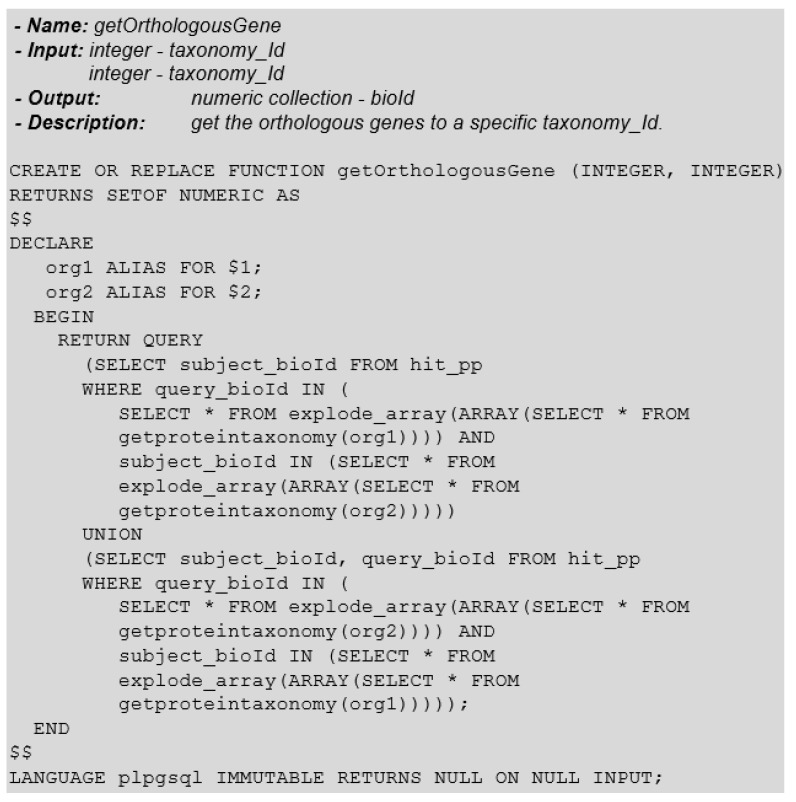
Function: GetOrthologousGene.

The function that returns orthologous genes is similar to the *getSimilarProtein* function. The difference is related to the type of returned information. In Figure 18 the function that returns orthologous genes of a certain species, concerning the other species, is presented, simply informing each species’ taxonomy-id.

We can also extend this function by adding input parameters to restrict the universe of selected data, such as hit-pp parameters, and reduce the search to a subset of genes. Likewise, we can enrich the output data with information about orthologous genes.

In paralogy, the objective is to identify proteins from a complete proteome of a given organism that is similar to other proteins of the same proteome. In this case, the search must start from the “root” of the taxonomic node, receiving the taxonomy-id in the same way that we did for single genes. Again, for this taxonomy-id we must consider all genomic sequences from the Genomic Sequence entity that corresponds to the origins of proteins compared to the research project to obtain the proteins.

**Figure 19:**
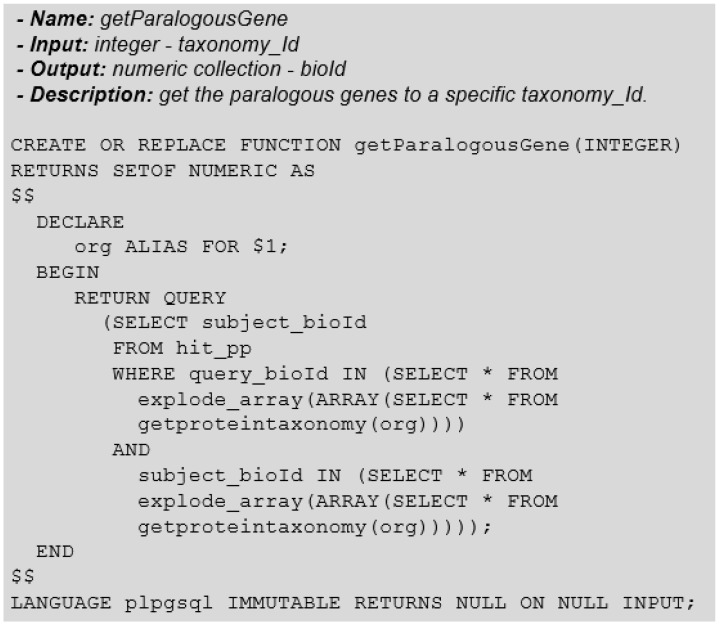
Function: GetParalogousGene.

Due to the abstraction present in our modelling, which presents the taxonomy-id in proteins, we can obtain the set of proteins without having to go through the genomic sequences. Finally, we must look in the hit-pp table for the existence of similarity between the proteins in this proteome. Figure 19 shows the function for obtaining similar genes.

Similarly, as for orthologous genes, the function of parallel genes can be extended by adding input parameters to restrict the universe of selected data, such as hit-pp parameters.

## 4. Discussion

We may find in the literature a larger interest in conceptual models, rather than logical or even physical models, for genomics and the related data. The authors in [10] suggest the idea of a genome data model and propose a representation for biological sequences based on arrays of sequence (strings) compositions, or even trees of partial sub-sequences. It is a quite complex way to represent sequences with no proper functions. Many other authors focus on the conceptual modeling issues, e.g., [11,12,13] that do not have a direct correspondance with logical and implemented data representations.

The authors in [14] discuss if complex data structures and data types concerning the biological domain are not well supported in most database systems. They mention the idea of user-defined data types in relational database systems. However, the implementation of sequences is based on BLOBs referred by generic tables. One of our previous works [15] deals with conceptual modeling with a focus on UML constructs and next-prior types for building complex data structures. Semantics were left exclusively for GeneOntology search.

The idea of a sequence-centric database schema is discussed in [16]. The authors discuss an approach that focuses on implementing strings but with no associated semantics. A similar strategy was adopted in [17], where the authors consider relational databases for persisting *short-reads*. Even if they show good performances for experimental results, no biological interpretation is present.

We may also cite a relatively old but still interesting research work [18] that wanted to make some comparisons with the technology then available for relational and object-oriented database systems. One of the main problems was related to table normalization issues. They use a single column, called sequence, to store short-reads. No further details are given concerning space limitations and string interpretation. More recently, the authors in [19] discuss the relational database approach and SQL queries for genomic datasets. An ad-hoc data format is considered, but the main goal refers to query distributed processing. The authors even explain that their idea was to separate genomic information from its underlying representation. Therefore, there is very little consideration on the actual sequence implementation.

Considering that a relational database system is available, some index structures for manipulating sequences have been proposed, especially the suffix tree [20,21]. It is a versatile and very efficient data structure built in linear time if it can be stored in main memory [22]. The authors in [23] are mainly concerned with the evaluation of relational and non-relational (NoSQL) implementations for genomic data. However, data representation and semantic associations are neither presented nor discussed.

### 4.1. Experimental Results

Besides efficacy and expressiveness with our *bio-string* ADT, we have implemented the related functions to study performance and space issues regarding our proposal. Even if our main goal here is to advocate in favor of modeling very large biological sequences as an adapted text (string) data type, it is crucial to evaluate the behavior in practice.

We have considered the following software and hardware configurations: a Linux (Debian distribution) operating system and PostgreSQL DBMS version 11; a virtual machine inside our Academic Laboratory internal cloud with four vCPUs i7-based and 32 GB RAM, besides 4TB of non-redundant storage.

To carry out some experimental tests related to the stored functions proposed and developed in this paper, we had to deal with importing relevant data from different external sources:

- Comparison data (hits) from the genome comparison project in partnership with World Community Grid [24];
- NCBI Taxonomy;
- Central Dogma information from the NCBI Reference Sequence (RefSeq);
- Annotations from the following sources:
  – the domain (Pfam);
  – enzymes (KEGG);
  – ontologies (Gene Ontology).

We developed scripts in *Perl* language to help with the ETL procedure into the relational database. After loading the data about proteins, genomic sequence, CDS, and taxonomy, our *hits* were loaded, summing up into the relational physical storage space about 360 GB (compressed data). These data are organized initially into over 900 files in TAR (packaged) format. Each file is a list of 2000 files in *.tar.gz format.

For this case study, we considered only a subset of the similarity data. This subset includes only similarity data whose e-value is less than or equal to 1.0 × 10^−3^. We used this cut-off value because this is the assumed base similarity value between those proteins in the Protein World Database (PWD) research project [24]. Using this filter, the amount of data in the *hit_pp* table reduced from 900 GB to approximately 300 GB. Observe that data that refers to ORFs and ontology annotations were not loaded. We have focused our search domain on proteins and the set of annotations to domains and enzymes.

Next, Table 1 presents the physical space occupied by some of the most relevant relations in our biological database.

We can observe at Table 1 that, in terms of physical space occupied, the largest table is *Hit_pp*, and then we have medium-sized relations like *CDS*, *Gene*, and *Genomic_sequence*.

### 4.2. Database Tuning

After loading the data, the implementation of some queries was started, as previously presented. For this initial set of tests, the relational database contained indexes for query optimization and processing and keys for referential integrity.

The use of indexes and keys (primary and foreign) are of great importance to help improve query performance and referential integrity of data, respectively. For this and other characteristics, many defend using DBMS to store and manage biological data since there are already consolidated mechanisms for data storage and management. However, the discussion ends up turning to the expressiveness of the existing types of data when used for the biological domain.

It is worth highlighting the process carried out to create indexes on the *hit_pp* table. Those queries involving similarity data always reference the identification attributes so, it was pretty straightforward to suggest indexes for these attributes, in addition to the fact of referencing the *protein* table. Another common tuning technique was the clustering of the *hit_pp* table based on these indexes. Summarizing the actions taken to build this case study testbed:

- database containing a subset of the *hit_pp* table;
- secondary indexes to speed up the performance on the *hit_pp* relation;
- *hit_pp* table clustered based on its indexes;
- all other tables only with primary keys (PK) indexes and unique constraints.

### 4.3. Data and Experiments

To carry out the tests, we used two organisms, both of the genus *Xanthomonas* (Taxonomy id: 338), which are (i) *Xanthomonas axonopodis* pv. *citri* str. 306 chromosome, complete genome, with gbkid = NC_003919.1; taxonomy id = 190486; GI = 21240774; and Refseq Protein = 4427 and (ii) *Xanthomonas campestris* pv. *campestris* str. ATCC 33913, complete genome; gbkid = NC_003902.1; Taxonomy id = 190485; GI = 21229478; and Refseq Protein = 4179.

First, the so-called *basic* queries were executed on our input data, as they are only related to statistical data. Then we ran the *complex* queries. Table 2 presents a summary of the results obtained.

Analyzing the results shown in Table 2, we can see that we have observed an excellent performance. Even queries that reference the *hit_pp* table—about 300 GB—had satisfactory results. Indeed, there are no issues concerning the actual execution involving the implemented function based on our bio-string type. As we do for conventional relational applications, simple tuning techniques are enough to speed up the running queries, even the most complex ones.

## 5. Conclusions

We have proposed in this paper to explore the relational text type already present in relational DBMS, and to model and implement extensive biological sequences. With the help of a set of domain-oriented functions, we may manipulate and extract biological information based on a given relational data schema. Available solutions either in the literature or included in specific software (i) do not explicitly explain the way they deal with sequences, (ii) do not make those extensions that add biological semantics to regular text strings or included in specific software, (i) do not explicitly explain the way they deal with sequences, and (ii) do not make those extensions that add biological semantics to regular text strings.

We have also discussed a *generic* biological conceptual schema that helps reinforce some biological concepts, regardless of specific research projects. We have shown that an actual implementation of sequence-oriented functions on *bio-strings* type is feasible and effective. The set of proposed rules and functions maps the intrinsic semantic information from the very long character sequence representing biological concepts.

Our implementation also shows that it is quite feasible to deal with biological data within a relational database system. The relational model is not a problem but, rather, the lack of semantics in existing data structures and types.

Our next steps include the idea of exploring other possible Abstract Data Type for the biological domain. We look forward to enabling this extension as a PostgreSQL DBMS *contrib* publicly available. We invite the reader to check and evaluate our current implementation and PostgreSQL PLPGSQL code at https://github.com/sergiolif/BioBD_SGBDBio, accessed on 17 June 2022. For relational DBMSs other than PostgreSQL, those particular SQL plus host language programming codes may be slightly different.

## Figures and Tables

**Table 1:** PWD relations.

Table	Size
CDS	617 MB
Domain	936 KB
Domain Annotation	1.2 MB
Enzyme Annotation	9.1 MB
Gene	512 MB
Genomic Sequence	413 MB
Hit_pp	288 GB
Protein	1.3 MB
Taxonomy	60 MB

**Table 2:** Experimental Results.

Query	Results	Time (ms)
Number of Compared Proteins	1,947,724	985.29
Proteins from Genomic Sequence	8,744,479	2359.13
Genomes belonging to a Taxonomic Group (id: 338)	22	19,888
Proteins belonging to a Taxonomic Group (id: 338)	42,923	39,116
Number of hits for Protein X cut-off e-value 1.0 ×10^−5^	3	3637
Unique Genes (taxonomy ID: 190485 190486)	585	5761.09
Orthologs Genes (taxonomy ID: 190485 190486)	3806	5862.71
Paralogs Genes (taxonomy ID: 190486)	12,655	3450.37

## Data Availability

https://github.com/sergiolif/BioBD_SGBDBio, accessed on 17 June 2022.

## Abbreviations

The following abbreviations are used in this manuscript:

DNA	deoxyribonucleic acid
DBMS	database management system
RDBMS	relational database management system
ADT	abstract data types
BLOB	binary large objects
A	adenine
T	thymine
C	cytosine
G	guanine
RNA	ribonucleic acid
U	uracil
ORF	open reading frame
Met	methionine
URF	unidentified reading frame
CDS	coding sequence
GC content	guanine and cytosine content
RefSeq	reference sequence database of NCBI
SW score	Smith&Waterman score
KEGG	Kyoto Encyclopedia of Genes and Genomes
Pfam	Protein Family Database
GO	Gene Ontology Resource
PLPGSQL	Programming Language PostgreSQL
CPU	Central Processing Unit
RAM	Random Access Memory
TB	Terabytes
GB	Gigabytes
NCBI	National Center for Biotechnological Information
ETL	Extract, Transform and Load
PWD	Protein World Database
PK	Primary Key
